# From Plants to Protection: Cardiorenal Benefits in Non-Diabetic Chronic Kidney Disease and Heart Failure

**DOI:** 10.3390/molecules30193982

**Published:** 2025-10-04

**Authors:** Dan Claudiu Măgureanu, Raluca Maria Pop, Ioana Corina Bocsan, Maria Adriana Neag, Angela Cozma, Antonia Mihaela Levai, Veronica Sanda Chedea, Anca Dana Buzoianu

**Affiliations:** 1 Pharmacology, Toxicology and Clinical Pharmacology, Department of Morphofunctional Sciences, “Iuliu Hațieganu” University of Medicine and Pharmacy, Victor Babes, No 8, 400012 Cluj-Napoca, Romania; dan.clau.magureanu@elearn.umfcluj.ro (D.C.M.); raluca.pop@umfcluj.ro (R.M.P.); maria.neag@umfcluj.ro (M.A.N.); abuzoianu@umfcluj.ro (A.D.B.); 2 Department of Internal Medicine, “Iuliu Hațieganu” University of Medicine and Pharmacy, 400012 Cluj-Napoca, Romania; angelacozma@yahoo.com; 3 Obstetrics and Gynecology, Department of Mother and Child, “Iuliu Hatieganu” University of Medicine and Pharmacy, Victor Babeș, No 8, 400012 Cluj-Napoca, Romania; levai.antonia@yahoo.com; 4 Research Station for Viticulture and Enology Blaj (SCDVV Blaj), 515400 Blaj, Romania; chedeaveronica@yahoo.com

**Keywords:** natural compounds, inflammation, oxidative stress, non-diabetic chronic kidney disease, heart failure

## Abstract

Non-diabetic chronic kidney disease (ND-CKD) refers to the progressive and irreversible decline in kidney function occurring in the absence of diabetes mellitus—a distinction that sets it apart from the more prevalent diabetic CKD. While diabetic nephropathy remains the leading cause of CKD globally, ND-CKD encompasses a heterogeneous group of etiologies, including hypertensive nephrosclerosis, glomerulonephritis, and interstitial nephritis. Its incidence and prevalence are steadily increasing, particularly in aging populations, and are often underrecognized. Importantly, ND-CKD is not a benign entity; it independently contributes to systemic inflammation, oxidative stress, and metabolic dysregulation, which in turn amplify cardiovascular risk. Among the most severe complications is heart failure (HF), a complex syndrome arising from structural and functional impairments in cardiac performance. Despite ongoing advancements in HF management, mortality remains unacceptably high, ranging from 2–3% at 30 days to up to 50–75% over five years. Standard pharmacologic therapies frequently fall short in halting disease progression and may provoke undesirable side effects. This therapeutic gap has spurred growing interest in natural compounds with multi-targeted effects. Bioactive molecules such as arjunolic acid, kaempferol, luteolin, and resveratrol have shown anti-inflammatory and antioxidant properties that may offer dual benefits for both renal and cardiac function. By modulating shared molecular pathways—including those involved in inflammation, oxidative damage, and cellular dysfunction—these agents hold promise as adjunctive treatments in ND-CKD complicated by heart failure.

## 1. Introduction

Chronic kidney disease (CKD) has become a significant public health issue worldwide, with its incidence and prevalence increasing steadily in recent years. Research highlights the considerable impact of this disease, indicating that around 700 million people globally [1], or 8–16% of the population, suffer from CKD [2]. The disease is linked to high morbidity and mortality rates, with approximately 1.5 million deaths each year. The rising costs of CKD treatment [3], primarily driven by the growing need for kidney transplants for patients with end-stage CKD, further exacerbate this alarming trend [4]. CKD is characterized by a gradual and irreversible deterioration in kidney function due to structural and/or functional abnormalities [5]. While diabetes is a leading cause of CKD [6,7,8,9,10], a significant proportion of cases occur independently of diabetes and are classified as non-diabetic chronic kidney disease (ND-CKD). ND-CKD refers to CKD arising from non-diabetic etiologies such as hypertensive nephrosclerosis [11,12,13], obesity- and metabolic syndrome-related nephropathy [14,15,16,17,18], autoimmune diseases including glomerulonephritis and tubulopathies, and drug-induced nephrotoxicity, notably from chronic use of non-steroidal anti-inflammatory drugs [19]. This variability underscores the diverse etiological spectrum of ND-CKD and the influence of coexisting medical conditions, treatments received, and environmental factors that may contribute to disease progression [20]. Notably, ND-CKD carries a high risk of complications, especially cardiovascular, which remain a leading cause of morbidity and mortality in this population.

Heart failure (HF) is a multifaceted syndrome arising from various structural or functional changes in the heart, resulting in diminished pumping ability [21]. This condition is categorized based on the left ventricular ejection fraction (LVEF) into reduced, moderately reduced, and preserved ejection fraction types. Heart failure with reduced ejection fraction (LVEF below 40%) or moderately reduced ejection fraction (LVEF between 40% and 50%) is frequently triggered by hypertension, different valvular heart diseases, and ischemic heart disease. It is characterized by impaired or slightly impaired systolic function of the left ventricle [22]. Conversely, heart failure with preserved ejection fraction (LVEF over 50%) is commonly multifactorial but presents clinically similarly to heart failure with reduced ejection fraction. However, it is marked by increased ventricular stiffness and decreased left ventricular compliance, leading to impaired diastolic filling and reduced cardiac output [23]. This reduction in cardiac output can ultimately compromise renal function [24].

Throughout history, traditional medicine has utilized the power of natural compounds derived from plants, animals, and minerals for healing. These natural products laid the foundation for early medicine, offering a vast array of remedies for various ailments [25]. Modern science is now re-examining these natural compounds, unlocking the secrets behind their effectiveness. Thanks to advancements in biochemistry, researchers have been able to accurately characterize the ubiquitous natural compounds found in plants that were used for therapeutic purposes in the past. As a result, similarities have been observed between the effects of these natural compounds and those of first-line drugs in various chronic pathologies, such as heart failure and chronic kidney disease. Furthermore, natural compounds can serve as blueprints for designing synthetic drugs with enhanced therapeutic properties [26,27,28,29,30]. Importantly, the selection of natural compounds for this study, arjunolic acid, kaempferol, luteolin, and resveratrol, was based on existing evidence from experimental models and clinical research specifically focused on non-diabetic CKD and heart failure. These compounds have demonstrated anti-inflammatory and antioxidant effects in preclinical studies targeting pathways implicated in ND-CKD and HF, including oxidative stress, endothelial dysfunction, myocardial remodeling, and tubulointerstitial inflammation. Their pleiotropic mechanisms may confer both renoprotective and cardioprotective benefits in this complex clinical setting.

In view of the increasing global burden of ND-CKD and HF, with chronic kidney disease projected to rise from 23rd in 2022 to among the top 10 causes of disease burden worldwide by 2050, while heart failure is expected to remain the leading contributor [31], and considering the limitations of current pharmacological therapies, this study aims to evaluate the potential benefits of these natural agents. Our objectives are to assess their capacity to modulate inflammatory and oxidative pathways, preserve renal and cardiac function, and potentially delay disease progression, while minimizing the risk of adverse effects. This research responds to an urgent need for safe and effective adjunctive therapies for non-diabetic individuals suffering from chronic kidney disease and heart failure.

## 2. Search Strategy and Compound Selection

We conducted a comprehensive literature search using PubMed, Scopus, and Web of Science to identify studies published between 2005 and 2025 that investigated natural compounds with potential benefits in non-diabetic chronic kidney disease (ND-CKD) and heart failure (HF). Search terms included combinations of keywords such as “non-diabetic chronic kidney disease,” “heart failure,” “natural compounds,” “flavonoids,” “polyphenols,” “antioxidant,” “anti-inflammatory,” and the names of specific molecules (e.g., “resveratrol,” “kaempferol,” “luteolin,” “arjunolic acid”). Boolean operators (AND, OR) were applied to refine results. Inclusion criteria comprised original experimental studies, clinical data, and reviews published in English that addressed the effects of natural compounds on relevant pathophysiological pathways, namely oxidative stress, inflammation, fibrosis, and apoptosis, in both renal and cardiac contexts. We excluded articles that focused exclusively on diabetes-related CKD models without relevance to ND-CKD or articles that did not mention how the CKD was induced. Reference lists of key articles were manually screened to identify additional sources. Priority was given to studies providing mechanistic insights into molecular pathways (e.g., NF-κB, TGF-β, mitochondrial function), histopathological outcomes, or functional parameters such as glomerular filtration rate or left ventricular ejection fraction. Based on these criteria, only four compounds, arjunolic acid, kaempferol, luteolin, and resveratrol, fulfilled the dual requirement of demonstrating protective effects in both ND-CKD and HF models. Their consistent antioxidant, anti-inflammatory, antifibrotic, and antiapoptotic properties, along with evidence of functional improvements in preclinical and limited clinical studies, informed their selection for detailed discussion in this review.

## 3. Non-Diabetic Chronic Kidney Disease and Heart Failure-Biomarkers of Diagnostic and Prognostic

### 3.1. Non-Diabetic Chronic Kidney Disease

Non-diabetic chronic kidney disease (ND-CKD) refers to a progressive and often irreversible decline in renal function not caused by diabetes, but by a range of other etiologies such as hypertension, glomerulonephritis, chronic interstitial nephritis, nephrosclerosis, or exposure to nephrotoxic agents. As with all forms of CKD, ND-CKD is defined by persistent alterations in kidney structure or function lasting more than three months, including a decreased estimated glomerular filtration rate (eGFR below 60 mL/min/1.73 m^2^), albuminuria, proteinuria, active urinary sediment, structural or histopathological abnormalities, or a history of kidney transplantation [32]. This condition contributes significantly to the global burden of disease due to its association with increased risks of hospitalization, cardiovascular complications, cognitive impairment, and overall morbidity and mortality [33].

ND-CKD is staged using the same criteria as general CKD, based on the severity of kidney damage and functional decline. The two primary markers used in clinical practice are eGFR and albuminuria [34]. eGFR serves as a practical measure of the kidneys’ ability to filter waste products [35], while the urinary albumin-to-creatinine ratio (UACR) quantifies the extent of albuminuria, which independently predicts not only the rate of renal function decline but also the cardiovascular risk in these patients [36]. As ND-CKD advances, patients may experience worsening hypertension, anemia, and electrolyte disturbances, with the terminal stage requiring renal replacement therapy through dialysis or transplantation. Early identification and management of albuminuria in ND-CKD are crucial, yet underdiagnosis remains a significant issue. For example, a study by Chu et al. (2023) revealed that approximately 66% of hypertensive and diabetic patients with clinically significant albuminuria were overlooked due to insufficient screening [37]. This observation underscores the need for routine assessment of UACR and eGFR, even in patients without diabetes, to enable earlier intervention.

**Figure 1:**
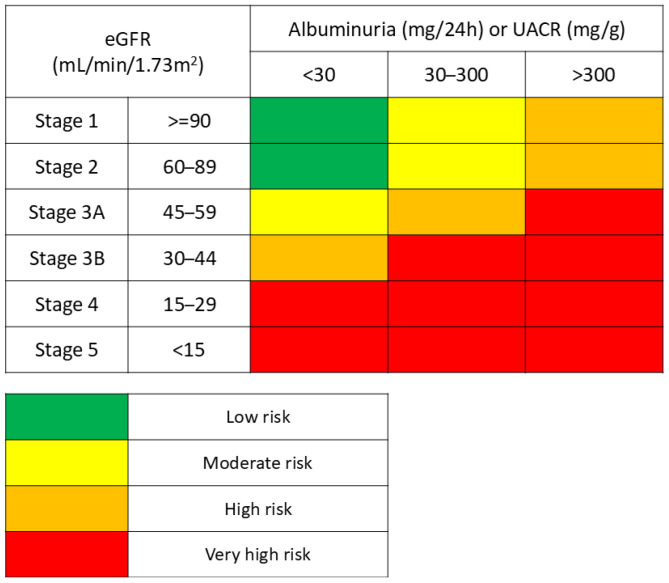
Risk of renal impairment based on eGFR and Albuminuria/URAC (adapted after “KDIGO 2024 Clinical Practice Guideline for the Evaluation and Management of Chronic Kidney Disease”).

Recognizing the clinical significance of both eGFR and albuminuria in predicting CKD progression, the KDIGO CKD Work Group developed a structured classification system that integrates these parameters to guide risk stratification and clinical decision-making [32,38] (Figure 1). This framework allows for individualized assessment of disease progression risk and supports tailored therapeutic approaches for patients with ND-CKD.

The progression of ND-CKD is heterogeneous and depends on multiple factors, including underlying pathology, comorbidities, genetic predisposition, and response to therapy. While the KDIGO staging system offers a valuable tool for stratifying patients, it does not capture this complexity entirely. Therefore, personalized management strategies, incorporating early diagnosis, close monitoring, and multidisciplinary care, are essential to slowing disease progression and improving patient outcomes. Early detection through routine screening remains one of the most effective strategies for altering the course of ND-CKD and minimizing long-term complications. However, for many CKD patients, while a complete cure may not be achievable, therapeutic interventions can significantly slow the progression of renal function decline [39].

### 3.2. Heart Failure

Heart failure (HF) is a complex clinical syndrome characterized by a spectrum of underlying pathophysiological mechanisms [40]. These mechanisms include structural and functional abnormalities of the heart, which can impair the ability of the heart to pump blood effectively or to fill adequately during diastole. The diverse etiology of heart failure necessitates a robust classification system to facilitate accurate diagnosis, effective risk stratification, and the development of targeted therapeutic strategies [41].

This refined classification system provides a framework for understanding the diverse etiological landscape of heart failure, paving the way for targeted therapeutic approaches.

Effectively managing heart failure necessitates a precise assessment of a patient’s functional capacity. The New York Heart Association (NYHA) classification system [42], a cornerstone in clinical practice, stratifies patients based on their ability to tolerate physical exertion and the severity of symptoms experienced during activity, ranging from no limitations (Class I) to severe symptoms even at rest (Class IV) [43].

The NYHA classification serves as a pivotal determinant of therapeutic strategies in heart failure management. For patients exhibiting mild to moderate symptoms (NYHA Class I and II), treatment is primarily aimed at symptom alleviation, quality of life enhancement, and disease progression prevention. This involves meticulous medication optimization, lifestyle interventions, and patient education, collectively improving functional capacity. Conversely, patients with severe symptoms (NYHA Class III and IV) require a more aggressive approach focused on maximizing functional capacity and symptom control to preserve independence and well-being. This encompasses medication adjustments, comprehensive symptom management, and potentially, cardiac rehabilitation [44,45,46].

Another classification criterion of heart failure is based on the left ventricular ejection fraction (LVEF), which serves as a critical determinant in stratifying heart failure patients. Heart failure with reduced ejection fraction (HFrEF), characterized by an LVEF ≤ 40%, reflects impaired systolic function leading to reduced cardiac output [22]. In contrast, heart failure with preserved ejection fraction (HFpEF), with an LVEF ≥ 50%, is associated with diastolic dysfunction and impaired ventricular filling [47]. The distinct pathophysiologies of these two phenotypes necessitate tailored therapeutic approaches. However, the emergence of heart failure with moderately reduced ejection fraction (HFmrEF), a category encompassing LVEF values between 41 and 49%, underscores the spectrum of heart failure and the need for more nuanced treatment strategies [48].

Heart failure represents a complex constellation of factors rather than a singular disease. A comprehensive understanding of its underlying etiology, a patient’s functional capacity, and the heart’s pumping ability is essential for optimal care. This individualized approach enables tailored treatment strategies. As scientific knowledge expands, the classification of heart failure will undoubtedly evolve, leading to more precise diagnoses, effective therapies, and improved patient outcomes.

### 3.3. Biomarkers in Non-Diabetic Chronic Kidney Disease and Heart Failure: Interplay and Interpretation

In the management of patients with non-diabetic chronic kidney disease (ND-CKD) and heart failure (HF), biomarkers play a central role in diagnosis, risk stratification, and prognosis. While estimated glomerular filtration rate (eGFR) and urine albumin-to-creatinine ratio (UACR) remain the primary measures for assessing kidney function [38], several circulating biomarkers are widely used in HF, including natriuretic peptides [49], troponins [50], galectin-3 [51], soluble ST2 (sST2) [52], and inflammatory markers such as C-reactive protein [53]. The interpretation of these biomarkers is strongly influenced by renal function, and conversely, cardiac dysfunction can impact renal biomarkers, reflecting the complex cardio-renal interplay [54,55]. Biomarkers in HF and ND-CKD can be broadly conceptualized based on their clinical utility as either diagnostic or prognostic. Diagnostic biomarkers help establish the presence of disease or acute decompensation. In HF, natriuretic peptides (BNP and NT-proBNP) are the most widely used, reflecting myocardial wall stress [49], while in ND-CKD, serum creatinine, cystatin C, and UACR indicate impaired renal function or structural kidney damage [38]. Prognostic biomarkers provide information about future risk, disease progression, or adverse outcomes. In HF, troponins, galectin-3, sST2, and inflammatory markers such as high-sensitivity C-reactive protein are primarily employed for risk stratification [51], whereas longitudinal trends in eGFR, cystatin C, and albuminuria predict ND-CKD progression and cardiovascular complications [38]. Renal function has a significant impact on the interpretation of HF biomarkers. Natriuretic peptides are cleared via the kidneys, and baseline levels are often elevated in ND-CKD independently of acute HF, necessitating adjusted diagnostic thresholds and careful trend monitoring [54]. Troponins can also remain chronically elevated in ND-CKD due to reduced clearance and subclinical myocardial stress [56]. Similarly, galectin-3 may be elevated in renal impairment, potentially overestimating HF risk [57]. In contrast, sST2 is less affected by kidney function, making it a particularly reliable prognostic marker in patients with coexisting HF and ND-CKD [58].

Conversely, HF influences renal biomarkers. Reduced cardiac output and increased central venous pressure can impair renal perfusion, transiently elevating creatinine and cystatin C levels, which may mimic ND-CKD progression [59]. Therapies for HF, such as diuretics or renin–angiotensin–aldosterone system inhibitors, can also modify renal biomarker levels [60]. This bidirectional interplay underscores the importance of interpreting biomarkers in the context of both cardiac and renal status, rather than relying on single measurements.

Overall, an integrated approach, combining renal function measures with HF biomarker trends, and using markers with lower renal dependence when possible, enhances diagnostic accuracy and prognostic precision in patients with overlapping ND-CKD and HF.

## 4. Therapeutic Strategies and Limitations

The intricate interplay between heart failure and chronic kidney disease presents a formidable challenge for clinicians. The heterogeneous etiologies and complex pathophysiological interactions of these conditions create a multifaceted clinical picture [61]. Individual patient variability, influenced by a wide range of comorbidities, further complicates therapeutic decision-making. The cardiovascular manifestations in patients with kidney dysfunction diverge from those observed in the general population, with atherosclerosis, cardiomyopathies, and congestive heart failure complications emerging as primary drivers of mortality [62]. Recognizing the profound impact of kidney function on heart failure prognosis, therapeutic strategies that mitigate renal deterioration are paramount. Notably, medications that concurrently enhance renal perfusion and cardiac output have shown substantial benefits in this patient population, underscoring the importance of a holistic approach to management [49] (Table 1).

Despite the availability of various therapeutic classes, establishing an optimal treatment regimen remains highly challenging, especially in patients suffering from both heart failure and chronic kidney disease, an increasingly common combination. For instance, beta-blockers have not demonstrated direct efficacy on renal function in chronic kidney disease and are only recommended in the presence of concomitant heart failure [64]. Angiotensin-converting enzyme inhibitors (ACEIs) have proven effective on renal function in hypertensive disease, while angiotensin receptor blockers (ARBs) are considered second-line therapy to replace ACEIs in cases of hypersensitivity or other contraindications [77]. Regarding diuretics, this therapeutic class has a very narrow therapeutic index in patients with advanced chronic kidney disease and congestive heart failure, as doses required to stabilize one condition often lead to the worsening of the other [49]. Lastly, SGLT2 inhibitors have demonstrated remarkable efficacy in heart failure and are now also indicated for chronic kidney disease regardless of diabetic status, although they are not recommended for patients with type 1 diabetes due to the risk of diabetic ketoacidosis [78]. Therefore, patients suffering from heart failure and chronic kidney disease, particularly non-diabetic ones, continue to suffer from a continuous deterioration of renal function. Thus, this subclass of patients requires additional therapies for a better quality of life. In this context, considering the extensive research conducted in recent years on natural compounds and the extremely promising initial results, therapies that can be proven effective for these patients could be developed based on them, rather than embarking on the path of synthesizing new molecules.

## 5. Natural Compounds

Throughout history, various natural therapies have been used to treat different pathologies, including chronic kidney disease and heart failure. These approaches often relied on herbal remedies, dietary modifications, and lifestyle changes. Traditional Chinese medicine, for example, employed herbs like dandelion root and licorice root to support kidney function and reduce inflammation [79]. Ayurvedic medicine advocated for a balanced diet and lifestyle to promote overall health and address underlying imbalances that could contribute to these conditions [80]. In ancient Greece and Rome, therapeutic baths and herbal infusions were used to treat kidney diseases and heart diseases [81]. In contemporary medicine, although the primary focus remains on the development of synthetic drugs, there is growing interest in naturally occurring compounds found in foods, including arjunolic acid, kaempferol, luteolin, resveratrol, and pterostilbene.

**Figure 2:**
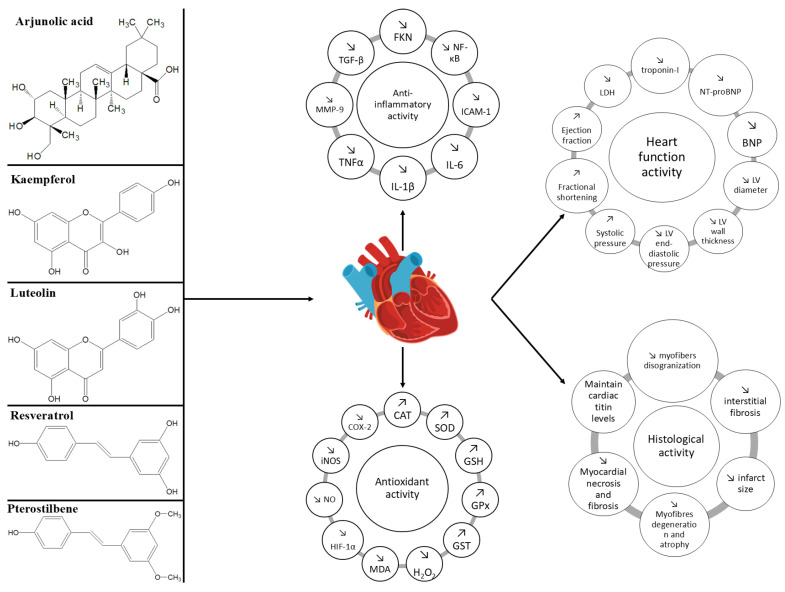
The cardioprotective activity of natural compounds.

**Figure 3:**
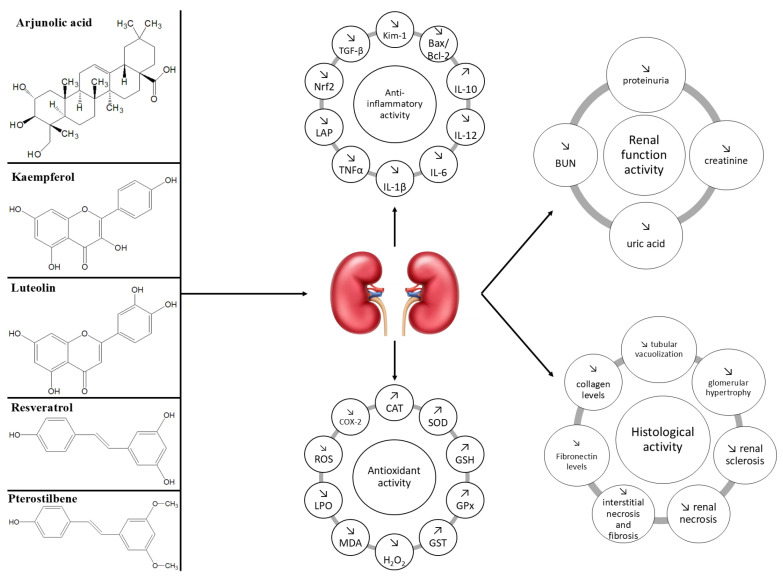
The renoprotective activity of natural compounds.

However, a significant limitation of the current literature is the predominance of diabetes-induced CKD models or the lack of clarity regarding the underlying etiology of CKD in experimental studies. In contrast, the present investigation specifically focused on identifying natural compounds that demonstrated beneficial effects in both heart failure and non-diabetic chronic kidney disease (ND-CKD). Only a limited number of bioactive agents, namely arjunolic acid, kaempferol, luteolin, and resveratrol, met this dual criterion, as they were the only compounds found in the literature to exhibit protective properties in models of ND-CKD as well as HF. These findings informed the selection of compounds evaluated in this study (Figure 2 and Figure 3), ensuring relevance to the targeted patient population and pathophysiological mechanisms.

### 5.1. Arjunolic Acid

**Figure 4:**
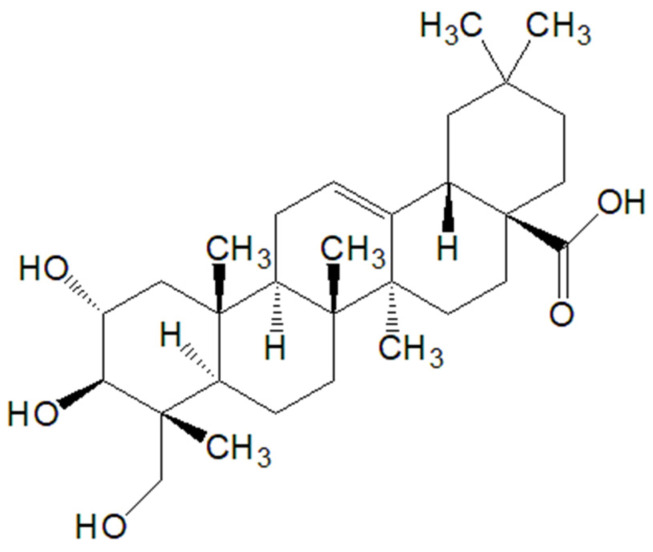
Chemical structure of arjunolic acid.

Arjunolic acid (Figure 4), a naturally occurring triterpenoid saponin, has garnered interest for its potential health benefits. Found in various plants used in Chinese traditional medicine like *Terminalia arjuna*, *Combretum nelsonii*, and *Lophostemon confertus*, arjunolic acid was investigated for its antioxidant, anti-inflammatory, cardioprotective properties, and, recently, for its renoprotective effects [82,83,84,85,86].

Therefore, in the study of Bansal et al. (2017), the researchers studied the impact of 10 mg/kg every other day of arjunolic acid on 21 male Wistar rats with right renal artery ligation-induced cardiac hypertrophy. They observed that the rats treated with arjunolic acid showed an improvement in cardiac function, a reduced expression of collagen, and a reduced activity of transforming growth factor β (TGF-β) [84]. When looking into the renoprotective effects of arjunolic acid, two studies that both used animal models of cisplatin-induced nephropathy observed that the rats treated with 100 or 250 mg/kg of arjunolic acid administered on the first, fourth, and seventh day of the experiment, respectively, with 20 mg/kg/day of arjunolic acid, had reduced levels of creatinine, urea, and blood urea nitrogen (BUN) and less histological signs of renal injury. Moreover, both studies ascribed the renoprotective effects to both antioxidant activity, noting markedly diminished levels of malondialdehyde (MDA), nitric oxide (NO), and latency-associated peptide (LAP), and concurrently increased levels of glutathione (GSH), alongside anti-inflammatory activity, demonstrated by lowered levels of nuclear factor-κB (NF-κB), tumor necrosis factor-alpha (TNFα), interleukin 1 beta (IL-1β), and kidney injury molecule-1 (Kim-1) in renal homogenates [85,86]. While further studies are needed to fully understand its therapeutic potential, arjunolic acid exemplifies the continued exploration of natural compounds that promote human health (Table 2).

### 5.2. Kaempferol

**Figure 5:**
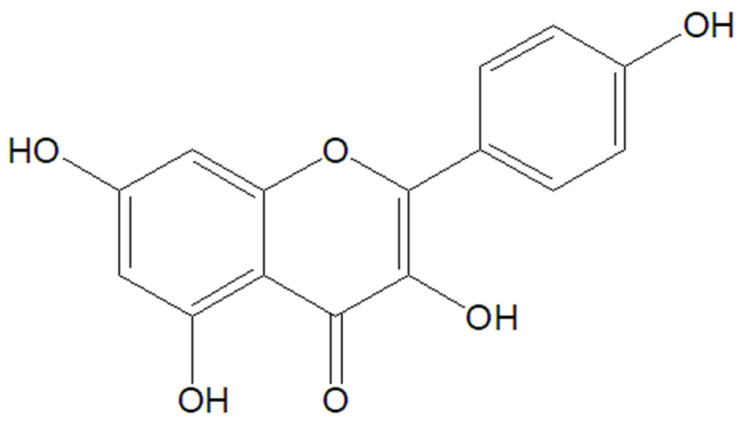
Chemical structure of kaempferol.

Kaempferol (Figure 5), a natural flavonoid, is widely distributed throughout the plant kingdom. Found in high concentrations in fruits, vegetables, and some medicinal herbs, it is particularly abundant in apples, grapes, kale, spinach, and green tea. Research suggests kaempferol possesses various health benefits, including antioxidant, anti-inflammatory, and potentially anticancer properties [87,88,89,90].

In addition to these effects and driven by the need for novel and effective therapies for heart failure and chronic kidney disease, researchers have conducted studies to investigate the cardioprotective and renoprotective effects of kaempferol (Table 3). Consequently, investigations were carried out on rats with isoproterenol-induced heart failure under the treatment with a daily dose of 10 or 20 mg/kg of kaempferol [91]. In this heart failure animal model, an enhancement of cardiac function was evident directly, as observed by diminished levels of brain natriuretic peptide (BNP), lactate dehydrogenase (LDH), and troponin I, as well as by reducing the levels of pro-inflammatory cytokines such as interleukin 6 (IL-6), interleukin 1 beta (IL-1β), tumor necrosis factor-alpha (TNF-α), while increasing the levels of the antioxidant enzymes like superoxide dismutase (SOD), catalase (CAT), glutathione reductase (GR), glutathione peroxidase (GPx), and glutathione-S-transferase (GST). Renoprotective effects were further corroborated in the investigation by Wang et al. (2020) involving rats with cisplatin-induced nephropathy and in the study by Guan et al. (2023) involving rats with hypertensive chronic kidney disease in both under treatment with kaempferol, in the first one using a daily dose of 100 or 200 mg/kg, while in the second study a daily dose of 10, 20 or 40 mg/kg. Both studies highlighted enhancements in renal function, as evidenced by both a reduction in serum creatinine and BUN levels and a reduction in histologically observed renal atrophy and necrosis [92,93].

### 5.3. Luteolin

**Figure 6:**
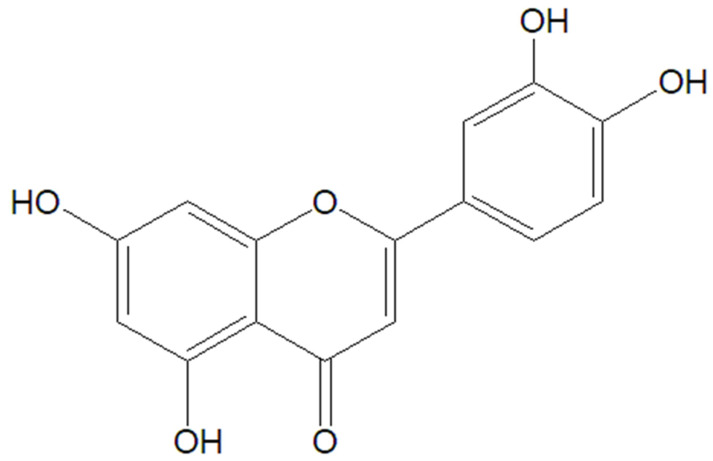
Chemical structure of luteolin.

Luteolin (Figure 6) is a flavonoid, a type of plant-based compound known for its antioxidant and anti-inflammatory properties. It was observed that luteolin possesses many potential health benefits, from anti-inflammatory and antioxidant activities to even neuroprotective properties [96,97,98,99,100,101].

Besides these, recent studies suggest a potential extension of the beneficial effects of this natural compound on the cardiovascular and renal systems. In this stance, the cardioprotective effects were investigated in rats with pressure overload via abdominal aortic constriction-induced heart failure under the treatment with luteolin using a daily intraperitoneal dose of 10 μg/kg [102]. In this heart failure animal model, the researchers observed that the treatment with luteolin decreased left ventricular diameters while increasing both ejection fraction and fractional shortening. Moreover, during the histological examination, they observed a reduction in myocardial apoptosis and fibrosis. Renoprotective effects were highlighted in the studies by Domitrović et al. (2013) and Arslan et al. (2016), where the researchers studied the effects of luteolin on rats with cisplatin-induced nephropathy, which was administered intraperitoneally using a dose of 10 mg/kg. Both research teams observed improvements in renal function, as indicated by lower serum creatinine and BUN levels, and reduced histological signs of renal injury, atrophy, and necrosis [103,104]. Consistent with the findings observed with arjunolic acid and kaempferol, luteolin also exhibited robust antioxidant properties in both animal models, as demonstrated by markedly elevated levels of glutathione reductase (GR), superoxide dismutase (SOD), and catalase (CAT), and anti-inflammatory properties, characterized by diminished levels of interleukins 12 and 1β, of tumor necrosis factor alpha (TNF-α) and a reduction in the expression of several pro-inflammatory genes such as tumor protein-53 (TP53), nuclear factor-κB (NF-κB), and Bax/Bcl-2. Studies are ongoing to elucidate its full therapeutic potential, but both kaempferol’s and luteolin’s presence in commonly consumed foods make these intriguing natural compounds for promoting human health (Table 4).

### 5.4. Resveratrol

**Figure 7:**
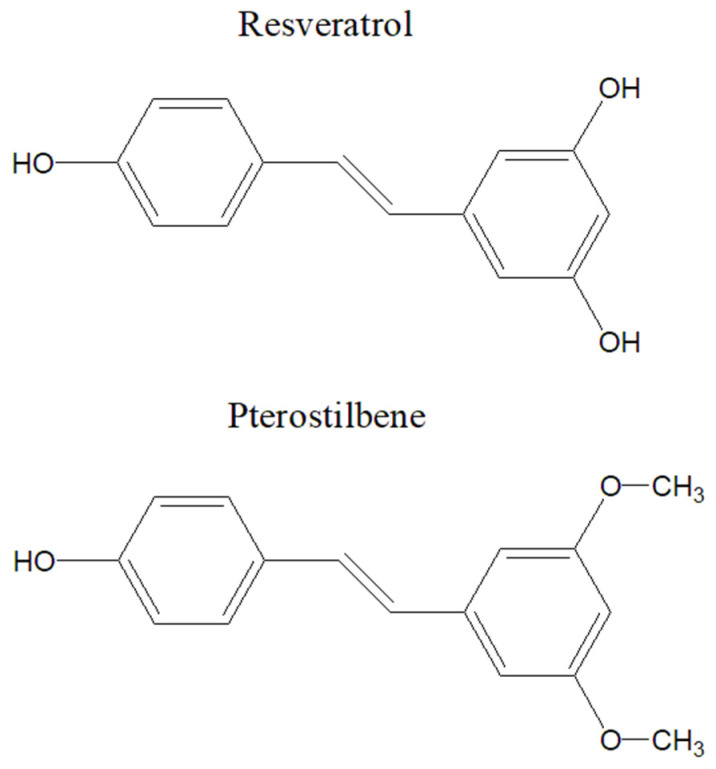
Chemical structures of resveratrol and pterostilbene.

Further expanding the repertoire of naturally occurring compounds with promising health benefits, resveratrol and pterostilbene (Figure 7) emerge as a subject of considerable scientific interest. Classified as a stilbenoid, resveratrol and, respectively, pterostilbene, a natural analog of it, are present especially in grapes, peanuts, and certain berries. While research into its therapeutic potential remains ongoing, resveratrol exhibits intriguing properties, including antioxidant and anti-inflammatory effects [110,111,112,113,114].

This investigation delves deeper into the current understanding of resveratrol, examining its potential applications within the medical field and emphasizing its contribution as a potential treatment for both heart failure and chronic kidney disease. In the context of heart failure, a significant study investigating the effects of resveratrol is that of Xuan et al. (2012) [115], who used an animal model of cardiac dysfunction induced by left coronary artery ligation. Their primary observation was that the intraperitoneal treatment with a daily dose of 20 mg/kg of resveratrol led to an enhanced survival rate. Furthermore, echocardiographic assessments revealed a reduction in heart chamber dimensions, coupled with an increase in ejection fraction and left ventricular shortening. These findings were attributed to a downregulation in the expression of several profibrotic and proapoptotic genes, including procollagen-1, procollagen-3, fractalkine, and matrix metalloproteinase-9 [115]. Similar echocardiographic observations were reported in the study by Raj et al. (2021), which examined the effects of daily resveratrol administration at a dose of 2.5 mg/kg [116], and in the study by Gupta et al. (2014), which utilized a dose of 10 mg/kg/day of resveratrol in an animal model of heart failure induced by aortic constriction [117]. While the full implications of inflammation and oxidative stress in heart failure are yet to be fully elucidated, studies such as that of Ma et al. (2023) have observed that a daily dose of 45.51 mg/kg in a rat model of angiotensin-II-induced cardiomyopathy exhibits anti-inflammatory effects, evidenced by a significant reduction in nuclear factor-κB (NF-kB) gene expression [118]. Similarly, the study by Zhang et al. (2023) demonstrated the antioxidant effects of resveratrol when administered at a daily dose of 50 mg/kg to rats with heart failure induced by aortic constriction, as reflected by enhanced expression of superoxide dismutase (SOD), glutathione peroxidase 4 (GPX4), glutathione (GSH), and solute carrier family 7 member 11 (SLC7A11) genes [119]. A significant step forward was made by Gal et al. (2020), who investigated the effects of administering a daily dose of 100mg resveratrol to 60 patients with heart failure and reduced ejection fraction in a single-center, randomized, double-blind, placebo-controlled trial. They observed that clinically, the therapy with resveratrol improved the quality of life, respiratory parameters, and exercise capacity, biologically, it reduced NT-proBNP and galectin-3 levels, and echocardiographically, resveratrol improved the left ventricular contractile function. Additionally, resveratrol exhibited anti-inflammatory effects, demonstrated by a significant reduction in interleukin 1 and 6 levels [120]. Therefore, this study highlights the potential therapeutic benefits of resveratrol in patients with HFrEF. Further research is warranted to confirm these findings and to determine the optimal dosage and duration of resveratrol treatment.

In the context of chronic kidney disease, the effects of resveratrol have been investigated in studies such as that of Chander et al. (2006) [121], who administered 5 mg/kg/day of resveratrol to 50 male Wistar rats with 5/6 nephrectomy-induced chronic kidney disease. They observed an increase in survival rate, a reduction in proteinuria and serum levels of creatinine, BUN, and urea, as well as a reduction in glomerular sclerosis and interstitial fibrosis during histological examination [121]. Similar observations were made in the study by Wang et al. (2020), who administered 200 mg/kg/day of pterostilbene to 20 rats with a high-adenine diet-induced chronic kidney disease [122]. In addition to its antioxidant and anti-inflammatory effects in heart failure, resveratrol also has these effects in chronic kidney disease. This was observed in the study of Cheng et al. (2019) [123], where the research team administered 100 mg/kg/day of resveratrol to 30 C57BL/6 male rats with high-fat diet-induced nephropathy. They found that resveratrol treatment exhibited significant anti-inflammatory activity in the renal system, characterized by a decrease in interleukin-6 (IL-6) and tumor necrosis factor-alpha (TNF-α) levels and an increase in interleukin-10 (IL-10) levels in renal homogenates. Moreover, resveratrol also acted as an effective antioxidant, leading to a decrease in malondialdehyde (MDA) levels and an increase in T-SOD and GPx levels in renal homogenates [123]. Similar effects of resveratrol were observed in the study by Liang et al. (2014), where daily administration of 20 mg/kg of resveratrol to 24 C57BL/6J male rats with unilateral ureteral obstruction-induced nephropathy led to enhanced antioxidant activity evidenced by an increase in SOD levels and a decrease in 8-OHdG and MDA levels in renal homogenates, respectively, to a reduce in inflammation, demonstrated by a decrease in the expression of intercellular adhesion molecule 1 (ICAM-1), transforming growth factor beta (TGF-β), and TNF-α, and an increase in silent information regulator 1 (Sirt1) gene expression [124]. In the existing literature, we have identified a single study that investigated the simultaneous effects of resveratrol on both the heart and kidneys. Li et al. (2020) examined the effects of daily administration of 20 mg/kg of resveratrol to 12 rats with 5/6-nephrectomy-induced chronic kidney disease on renal and cardiac functions. In the renal system, they observed a decrease in serum creatinine and BUN levels and histologically a reduction in renal fibrosis, sclerosis, and necrosis. Cardiac effects included, echocardiographically, a decrease in cardiac hypertrophy and cavity dilatation, while histologically, they included a reduction in cardiomyocyte hypertrophy and perivascular and interstitial fibrosis [125]. These findings suggest that resveratrol’s antioxidant and anti-inflammatory properties may contribute to its protective effects in both heart failure and chronic kidney disease (Table 5).

### 5.5. Common and Distinct Mechanisms of Action and Effects of Natural Compounds in Non-Diabetic Chronic Kidney Disease

Natural compounds such as arjunolic acid, kaempferol, luteolin, and resveratrol exert significant biological effects in non-diabetic chronic kidney disease (ND-CKD) through overlapping mechanisms that converge on the modulation of oxidative stress, inflammation, and fibrotic remodeling (Table 6). In ND-CKD, these compounds have consistently demonstrated renoprotective effects by attenuating tubular damage, glomerular sclerosis, and interstitial inflammation [86,95,108,121,124]. These effects are largely mediated through the enhancement of endogenous antioxidant defenses, characterized by increased activity of enzymes such as superoxide dismutase (SOD), glutathione peroxidase (GPx), and glutathione-S-transferase (GST), alongside a reduction in lipid peroxidation markers such as malondialdehyde (MDA) and nitric oxide (NO) [109,113]. In addition to their antioxidative properties, these compounds also exert pronounced anti-inflammatory effects by down-regulating nuclear factor-κB (NF-κB) signaling and reducing the expression of pro-inflammatory cytokines, including tumor necrosis factor-alpha (TNF-α), interleukin-1 beta (IL-1β), and interleukin-6 (IL-6) [85,106]. These changes are associated with decreased leukocyte infiltration and preservation of renal parenchyma. Furthermore, several studies have highlighted the ability of these compounds to modulate apoptotic signaling pathways, with a notable down-regulation of pro-apoptotic markers such as p53 and Bax, and an up-regulation of anti-apoptotic proteins such as Bcl-2, thereby contributing to tubular and glomerular cell survival [86,92].

Another key mechanism involves the attenuation of fibrogenesis. Arjunolic acid and kaempferol have been shown to reduce transforming growth factor-beta (TGF-β) activity and the expression of extracellular matrix proteins, including collagen types I and III, which are hallmarks of renal fibrosis [85,93]. Similarly, resveratrol and luteolin have demonstrated the ability to decrease histological indicators of fibrosis and interstitial thickening. These effects were accompanied by improvements in renal function, evidenced by reductions in serum creatinine and blood urea nitrogen (BUN), as well as improved histological architecture in multiple experimental models of ND-CKD, including cisplatin-induced nephropathy, hypertensive nephropathy, and unilateral ureteral obstruction [103,124]. While these shared pathways underscore a unified mechanism of action, compound-specific differences also exist. For instance, kaempferol exhibited pronounced effects on calcium oxalate nephropathy, with evidence of decreased crystal deposition and oxidative burden [95]. Resveratrol, in turn, was associated with improved mitochondrial function and biogenesis, reflected in enhanced ATP synthesis and increased activity of respiratory complexes, particularly in nephrectomy-induced models of renal impairment [134]. Taken together, the available evidence supports the therapeutic potential of these natural compounds in ameliorating the pathophysiological processes underlying non-diabetic kidney injury through a combination of anti-inflammatory, antioxidant, anti-fibrotic, and cytoprotective actions.

### 5.6. Common and Distinct Mechanisms of Action and Effects of Natural Compounds in Heart Failure

In heart failure (HF), the pathophysiological mechanisms targeted by natural compounds align closely with those observed in chronic kidney disease, particularly concerning oxidative stress and inflammation. Arjunolic acid, kaempferol, luteolin, and resveratrol have been extensively studied in various animal models of heart failure, where they have demonstrated cardioprotective properties through the modulation of cellular redox state, cytokine signaling, and myocardial remodeling (Table 7).

A central mechanism shared across all compounds is their capacity to reduce oxidative damage within cardiac tissue. This is evidenced by elevated levels of endogenous antioxidants such as superoxide dismutase (SOD), catalase (CAT), and glutathione peroxidase (GPx) following treatment, along with a reduction in markers of oxidative injury, including malondialdehyde (MDA), nitric oxide (NO), and 4-hydroxynonenal (4-HNE) [91,106,116,119]. In parallel, anti-inflammatory effects have been consistently documented, with decreased expression of nuclear factor-κB (NF-κB) and pro-inflammatory cytokines, including tumor necrosis factor alpha (TNF-α) and interleukin 6 (IL-6), contributing to reduced myocardial inflammation and fibrosis [84,91,106,120]. Functionally, the administration of these compounds has been associated with improved cardiac performance. This includes increased left ventricular ejection fraction, reduced end-diastolic and end-systolic diameters, and attenuation of ventricular wall thickening [94,105,115,120]. Histological analyses have confirmed the reversal of adverse structural remodeling, as demonstrated by reduced collagen deposition, diminished myocyte hypertrophy, and preservation of cardiac architecture [94,102,128]. For example, arjunolic acid enhanced the expression of peroxisome proliferator-activated receptor alpha (PPARα), which plays a pivotal role in myocardial energy metabolism and contractility [84]. Similarly, luteolin was found to increase the expression of sarcoplasmic/endoplasmic reticulum Ca^2+^-ATPase 2a (SERCA2a), leading to improved calcium handling and contractile function [102]. Notably, some compounds exerted unique effects that extend beyond their shared antioxidative and anti-inflammatory properties. Resveratrol, in particular, influenced cardiac energy metabolism by activating adenosine monophosphate-activated protein kinase (AMPK) and sirtuin 1 (Sirt1), leading to enhanced mitochondrial efficiency and reduced cardiac fibrosis. These molecular effects translated into improved exercise capacity, reduced levels of natriuretic peptides, and improved quality of life in clinical settings [129,130]. Kaempferol also demonstrated efficacy in mitigating angiotensin II-induced myocardial damage, preserving both systolic and diastolic function [94].

In contrast to ND-CKD, where the focus lies primarily on preserving nephron integrity and preventing fibrotic progression, heart failure therapies aim to improve hemodynamics and reverse maladaptive myocardial remodeling. Nonetheless, the overlap in molecular targets across both conditions, particularly those involving oxidative and inflammatory pathways, supports the use of these compounds in the cardio-renal continuum. While further clinical studies are warranted, the current evidence suggests that natural compounds possess multimodal properties that may offer significant therapeutic advantages in the management of heart failure.

## 6. Knowledge Gaps and Future Directions

While the preclinical evidence supporting the therapeutic promise of natural compounds in non-diabetic chronic kidney disease (ND-CKD) and heart failure (HF) is both encouraging and substantial, it is equally important to acknowledge the current limitations that constrain their integration into clinical practice. A more complete understanding of their mechanisms, along with rigorous translational efforts, remains essential before these agents can be confidently adopted as adjunctive or standalone therapies.

One of the most significant limitations lies in the preclinical models themselves. Many experimental studies investigating natural compounds rely on acute, chemically induced forms of renal or cardiac injury, often in young, otherwise healthy animals. Such models, although informative, do not fully capture the complex, chronic, and multifactorial nature of ND-CKD and HF as they manifest in human patients, particularly older individuals with multiple comorbidities. Moreover, a disproportionate number of studies continue to focus on diabetic nephropathy, with far fewer specifically examining non-diabetic etiologies such as hypertensive nephrosclerosis, glomerulonephritis, or cardiometabolic-induced nephropathy. This distinction is critical, as the pathophysiological profiles and treatment responses may differ significantly between these subgroups. Additionally, although antioxidant and anti-inflammatory pathways have emerged as common targets of natural compounds, the precise molecular mechanisms remain only partially understood. While many studies identify reductions in oxidative markers or pro-inflammatory cytokines, fewer have explored the upstream regulators or downstream functional consequences of these changes. Critical signaling nodes, such as nuclear factor erythroid 2-related factor 2 (Nrf2), sirtuins, or mitochondrial transcription factors, are often under-investigated, and the tissue-specificity of these responses are not always clear. The interplay between systemic and local effects, particularly within the context of the cardio-renal axis, warrants deeper exploration through integrative, systems-level approaches.

Another area in need of development concerns pharmacokinetics and bioavailability. Several natural compounds, including resveratrol and kaempferol, are known to suffer from limited oral absorption and rapid metabolic degradation. Without appropriate delivery systems or bioenhancing strategies, their therapeutic potential may not be fully realized in vivo, especially in humans. Yet, formulation science has received limited attention in the current literature, and detailed pharmacokinetic profiles across species, disease states, and administration routes are still lacking. Furthermore, while preclinical findings are often promising, translation into human studies has been sporadic and, in many cases, absent. Clinical trials involving these natural agents remain limited in scale, scope, and methodological rigor. Resveratrol is perhaps the only compound among those discussed that has undergone meaningful clinical testing in heart failure patients, albeit with small sample sizes and relatively short durations. For the other compounds, there is a pressing need for controlled human studies to determine safety, tolerability, optimal dosing, and long-term efficacy. These trials should be designed to complement, not replace, existing standards of care, highlighting the role of natural compounds as supportive, rather than alternative, therapies. Finally, the potential for synergistic or antagonistic interactions between natural compounds and conventional medications has not been thoroughly studied. Patients with ND-CKD and HF frequently receive multiple pharmacologic treatments, and the introduction of natural agents could alter the pharmacodynamics or toxicity profile of these regimens. A more robust investigation into drug–nutrient interactions, especially in the setting of polypharmacy, would contribute greatly to patient safety and therapeutic efficacy.

Taken together, these gaps underscore the need for a multidisciplinary research agenda. Future studies should not only deepen mechanistic understanding but also address formulation challenges, incorporate realistic disease models, and include rigorous clinical trials. With such an approach, the therapeutic landscape for patients with ND-CKD and HF may be expanded in a manner that is both scientifically sound and clinically meaningful.

## 7. Conclusions

Non-diabetic chronic kidney disease and heart failure are increasingly prevalent conditions that share overlapping pathophysiological mechanisms and often coexist, thereby compounding disease burden and complicating management. Despite advances in pharmacological therapy, existing treatments remain limited in fully addressing disease progression, systemic inflammation, and oxidative stress. Preclinical and a few clinical findings reviewed here suggest that certain natural compounds, such as arjunolic acid, kaempferol, luteolin, and resveratrol, possess antioxidant, anti-inflammatory, antifibrotic, and antiapoptotic properties that may benefit both renal and cardiac systems. While their pleiotropic mechanisms point to potential value as adjunctive agents within the cardio-renal continuum, translation into clinical use is constrained by knowledge gaps, including a lack of disease-specific models, limited human data, poor bioavailability of some compounds, and incomplete understanding of pharmacodynamic interactions.

Future efforts should prioritize formulation improvements, mechanistic studies, and well-designed clinical trials to better define the role of these natural bioactives as complementary therapies alongside guideline-directed treatments. With further research, they may help expand therapeutic options for patients with non-diabetic chronic kidney disease and heart failure, but current evidence supports cautious optimism rather than substitution for established care.

## Figures and Tables

**Table 1:** Treatment options for heart failure and chronic kidney disease.

Therapeutic Class	Representants	Mechanism of Action	Therapeutic Outcomes	References
Beta Blockers	Bisoprolol Carvedilol Metoprolol	Beta-adrenergic blocking agents	CV *: Improve morbidity and mortality in chronic HFrEF by decreasing the heart rate and the contractility	[63]
			R **: Ameliorate renal function by reducing systemic vascular resistance–only in patients with heart failure	[64]
Inhibitors of Angiotensin Converting Enzyme (ACEI)	Lisinopril Captopril Perindopril Ramipril	Decreases the formation of angiotensin II	CV: Improve morbidity and mortality in chronic HFrEF by decreasing left ventricular afterload, leading to an increase in cardiac output and a decrease in heart-filling pressure	[65]
			R: Ameliorate renal function by reducing systemic vascular resistance	[66]
Angiotensin receptor blocker (ARB)	Candesartan Losartan Olmesartan Valsartan Irbesartan	Displaces angiotensin II from angiotensin I receptor	CV: Improve morbidity and mortality in chronic HFrEF by decreasing left ventricular afterload, leading to an increase in cardiac output and a decrease in heart-filling pressure	[67]
			R: Ameliorate renal function by reducing systemic vascular resistance	[68]
Angiotensin receptor blocker + Neprilysin inhibitor (ARNI)	Valsartan + Sacubitril	Displaces angiotensin II from angiotensin I receptor + prevents the breakdown of natriuretic peptides	CV: Improve morbidity and mortality in chronic HFrEF by preventing and reversing electrical and structural remodeling that appears in heart failure	[69]
			R: Prevent the progression of chronic kidney disease and lead to a need for lower doses of diuretic drugs	[70]
Mineralocorticoid receptor antagonist (MRA)	Spironolactone Eplerenone	Blocks mineralocorticoid receptors from the activation by aldosterone and 11-deoxycorticosterone	CV: Improve morbidity and mortality in chronic HFrEF by modulating the RAA system, leading to a reduction in ventricular remodeling	[71]
Non-steroidal mineralocorticoid receptor antagonist (nsMRA)	Finerenone	Selectively blocks mineralocorticoid receptors * differs from steroidal MRAs by a distinct cofactor-binding profile and lower risk of hyperkalaemia	CV: Improve morbidity in patients with HFrEF and diabetes or CKD by reducing hospitalization for heart failure through modulation of fibrosis and inflammation	[72]
			R: Ameliorate renal function by slowing the decline in eGFR	
Diuretics	Furosemide Bumetanide Indapamide	Blocks sodium reabsorption, which leads to an increase in urinary water and sodium losses	CV: Improve the morbidity in chronic HFrEF, HFmrEF, and HFpEF by improving the volume overload, especially in a decompensated state	[73]
			R: Ameliorate renal function by maintaining an effective diuresis	[74]
SGLT2 inhibitors	Dapagliflozin Empagliflozin	Blocks the activity of the sodium-glucose cotransporter 2 in the proximal renal tubules, thereby lowering the renal threshold for glucose	CV: Improve morbidity and mortality in chronic HFrEF, HFmrEF, and HFpEF by increasing natriuresis, promoting ketone production (alternative source of energy for cardiomyocytes), and blocking RAA system	[75]
			R: Prevent the progression of diabetic chronic kidney disease by better glycemic control	[76]

* The cardioprotective activity (CV) of therapeutic drugs. ** The renoprotective activity (R) of therapeutic drugs. Abbreviations: HFrEF—heart failure with reduced ejection fraction; HFmrEF—heart failure with moderately reduced ejection fraction; HFpEF—heart failure with preserved ejection fraction; RAA—renin–angiotensin–aldosterone.

**Table 2:** Effects of arjunolic acid on heart failure and non-diabetic chronic kidney disease.

Different Models of Heart Failure				
Dosage of Arjunolic Acid	Model	Anti-inflammatory and Antioxidant Effects	Beneficial Heart Effects	Reference
Arjunolic acid (10 mg/kg/ alternate day–intraperitoneal from 6th to 14th day)	21 male Wistar rats with ligated right renal artery-induced cardiac hypertrophy	- down-regulated TGF-β activity	- mitigated collagen-1 and collagen-3 expression - improved cardiac function - up-regulated PPARα expression	[84]
**Different Models of Non-Diabetic Chronic Kidney Disease**				
**Dosage of arajunolic acid**	**Model**	**Anti-inflammatory and antioxidant effects**	**Beneficial kidney effects**	**Reference**
Arajunolic acid (100 or 250 mg/kg–oral on 1st, 4th, 7th day)	50 male Sprague Dawley rats with cisplatin-induced nephropathy	- reduced TGF-β, MDA, and LAP levels in renal homogenate - reduced NADPH oxidase activity in renal homogenate - reduced NF-κB, TNFα, IL-1β, caspase-8 and caspase-9 levels in renal homogenate	- increased survival rate - significantly reduced plasma creatinine and urea levels - histological: mitigated dilatation of Bowman’s space, shrinkage of glomerular tufts	[85]
Arajunolic acid (20 mg/kg/day–oral for 10 days)	30 male Sprague-Dawley rats with cisplatin-induced nephropathy	- significantly reduced MDA and NO levels in renal homogenate - significantly increased GSH activity in renal homogenate - down-regulated TGF-β, Kim-1, and NF-κB expression in kidney tissue - up-regulated Bcl-2 expression in kidney tissue	- significantly reduced serum creatinine and BUN levels - histological: reduced tubular cell necrosis and degeneration	[86]

Abbreviation: PPARα—peroxisome proliferator-activated receptor α; TGF-β—transforming growth factor β; MDA—malondialdehyde; LAP—latency-associated peptide; NADPH—nicotinamide adenine dinucleotide phosphate; NF-κB—nuclear factor-κB; TNFα—tumor necrosis factor-alpha; IL-1β—interleukin 1 beta; BUN—blood urea nitrogen; NO—nitric oxide; GSH—glutathione; Kim1—kidney injury molecule-1; Bcl-2—B cell lymphoma-2.

**Table 3:** Effects of kaempferol on heart failure and non-diabetic chronic kidney disease.

Different Models of Heart Failure				
Dosage of Kaempferol	Model	Anti-inflammatory and Antioxidant Effects	Beneficial Heart Effects	Reference
Kaempferol (10/20 mg/kg/day–oral for 42 days)	60 male Wistar rats with isoproterenol-induced heart failure	- decreased MDA levels - increased CAT, GPx, SOD, GR, and GST levels - significantly decreased IL-6, IL-1β, TNF-α, and NF-κB levels	- significantly reduced LDH, BNP, and troponin-I levels - maintained systolic blood pressure - significantly increased Nrf-2, γ-GCS and HO-1 mRNA expression - significantly decreased Keap1 expression	[91]
Kaempferol (10 mg/kg/alternative day–oral for 28 days)	Male C57BL/6 rats with Angiotensin-II-induced heart failure	-	- mitigated decreases in ejection fraction and fractional shortening induced by Angiotensin-II - mitigated increases in diastolic dysfunction - reduced NT-proBNP levels - histological: reduced numbers of deranged cellular structures, disorganized myofibers; maintained cardiac titin levels	[94]
**Different Models of Non-Diabetic Chronic Kidney Disease**				
**Dosage of Kaempferol**	**Model**	**Anti-inflammatory and antioxidant effects**	**Beneficial kidney effects**	**Reference**
Kaempferol (100 or 200 mg/kg/day–oral for 14 days)	30 male Balb/C rats with cisplatin-induced nephropathy	- histological: decreased accumulation of inflammatory cells - both doses increased GR, SOD, and GST levels; the 200mg dose increased CAT, GSH, and NQO1 levels - decreased TBARS and iNOS levels - the 200 mg dose increased Nrf2 and HO-1 expression in renal tissue - down-regulated Bax/Bcl-2 and TP53 expression in renal homogenate - significantly reduced TNF-α and IL-12 levels in renal tissue - down-regulated JNK, p38, and ERK1/2 expression	- reduced serum creatinine and BUN levels - histological: decreased renal injuries	[92]
Kaempferol (25 or 50 mg/kg/day–oral for 10 days)	40 male C57BL/6 rats with calcium oxalate crystal-induced nephropathy	- down-regulated iNOS, NOX2, and NF-κB expression - decreased IL-6, IL-1β, and TNF-α levels - increased IL-4, IL-10, and Arg1 levels - reduced ROS, MDA, and H_2_O_2_ levels - increased SOD and GSH levels	- significantly reduced serum creatinine and BUN levels - histological: reduced formation of calcium oxalate crystal deposits in kidneys; reduced tubular damage	[95]
Kaempferol (10, 20, or 40 mg/kg/day–oral for 84 days)	25 male rats with spontaneously hypertensive-induced chronic kidney disease + 5 Wistar-Kyoto rats	- reduced MCP-1, TNF-α, and IL-1β levels - histological: reduced accumulation of inflammatory cells - reduced α-SMA levels in renal homogenate	- maintained blood pressure - reduced serum BUN and creatinine levels - reduced mALB level - down-regulation of TGF-β1, collagen I, and collagen III expression - histological: reduced tubular dilatation and atrophy	[93]

Abbreviation: MDA—malondialdehyde; CAT—catalase; SOD—superoxide dismutase; GR—glutathione reductase; GPx—glutathione peroxidase; GST–glutathione-S-transferase; LDH—lactate dehydrogenase; BNP—brain natriuretic peptide; NF-κB–nuclear factor- κB; TNFα—tumor necrosis factor-alpha; IL-1β—interleukin 1 beta; IL-6—interleukin 6; Nrf-2—nuclear factor erythroid 2 related factor 2; γ-GCS—γ-glutamylcysteine synthetase; HO-1—heme oxygenase 1; Keap1—Kelch-like erythroid cell-derived protein with CNC homology-associated protein 1; GSH—glutathione; BUN—blood urea nitrogen; NQO1—NAD quinone oxidoreductase 1; TBARS—thiobarbituric acid reactive substances; iNOS—inducible nitric oxide synthase; Bcl-2—B cell lymphoma-2; Bax—Bcl-2-associated X protein; Bcl-2—B-cell lymphoma 2; TP53—tumor protein-53; IL-12—interleukin 12; JNK—c-Jun N-terminal kinases; p38—protein 38; ERK1/2—extracellular-receptor kinases; BUN—blood urea nitrogen; NOX2— Nicotinamide Adenine Dinucleotide Phosphate Oxidase 2; IL-4—interleukin 4; IL-10—interleukin; Arg1—arginase-1; ROS—reactive oxygen species; mALB—microalbumin; MCP-1—monocyte chemoattractant protein-1; α-SMA—alpha smooth muscle actin.

**Table 4:** Effects of luteolin on heart failure and non-diabetic chronic kidney disease.

Different Models of Heart Failure				
Dosage of Luteolin	Model	Anti-Inflammatory and Antioxidant Effects	Beneficial Heart Effects	Reference
Luteolin (200 mg/kg/day–oral for 56 days)	40 male Sprague-Dawley rats with streptozotocin-induced diabetic cardiomyopathy	-	- increased left ventricular systolic pressure and left ventricular developed pressure - decreased left ventricular end-diastolic pressure - increased heart rate	[105]
Luteolin (10 μg/kg/day–intraperitoneal for 14 days)	91 male Sprague-Dawley rats with pressure overload via abdominal aortic constriction-induced heart failure	-	- up-regulated SERCA2a and Akt expression - decreased left ventricular internal diameters of end-systole and end-diastole - increased ejection fraction and fractional shortening - histological: reduced myocardium fibrosis and apoptosis	[102]
Luteolin (100 mg/kg/day–oral for 8 days)	32 male Wistar rats with cobalt chloride-induced cardiopathy	- reduced MDA, NO, and H_2_O_2_ levels - reduced MPO activity - increased GSH, GPx, and GST levels - down-regulated NF-κB expression in heart tissue	- histological: mitigated degeneration and atrophy of the myofibres	[106]
**Different Models of Non-Diabetic Chronic Kidney Disease**				
**Dosage of Luteolin**	**Model**	**Anti-inflammatory and antioxidant effects**	**Beneficial kidney effects**	**Reference**
Luteolin (50 mg/kg/day–oral for 3 days)	30 rats with ischemia–reperfusion-induced nephropathy	- significantly increased CAT, SOT, and GPx - reduced MDA levels in renal tissue - down-regulated miR320 and Nrf2 expression in renal tissue	- significantly reduced serum creatinine and BUN levels - histological: significantly mitigated renal injury	[107]
Luteolin (100 mg/kg/day–oral for 8 days)	32 male Wistar rats with cobalt chloride-induced nephropathy	- reduced MDA and H_2_O_2_ levels - increased GSH, GPx, and GST levels - down-regulated NF-κB expression in kidney tissue	- histological: mitigated tubular atrophy and nephron necrosis	[106]
Luteolin (10 mg/kg/day–intraperitoneal for 3 days)	30 male BALB/cN rats with cisplatin-induced nephropathy	- significantly reduced 3-NT and 4HNE levels in renal tissue - increased GSH level - down-regulated NF-κB, COX-2, and TNF-α expression in kidney tissue	- reduced serum creatinine and BUN levels - histological: mitigated tubular dilatation and tubular necrosis; down-regulated caspase-3 and p53 expression	[103]
Luteolin (10 mg/kg/day–intraperitoneal for 7 days)	28 male Wistar rats with cisplatin-induced nephropathy	-	- reduced serum creatinine level - histological: mitigated renal injury	[104]
Luteolin (100 or 200 mg/kg/day–oral for 28 days)	28 male Wistar rats with bisphenol A-induced nephropathy	- reduced serum IL-6, IL-1β, and TNF-α levels - reduced MDA level in renal tissue - increased SOD, GPx, and GSH levels in renal tissue	- reduced serum creatinine, BUN, and uric acid levels - histological: mitigated renal injury such as glomerular hypertrophy, epithelial cell edema, and accumulation of inflammatory cells	[108]
Luteolin (50 or 100 mg/kg/day–oral for 14 days)	50 male Wistar rats with doxorubicin-induced nephropathy	- increased SOD, CAT, GPx, GST, GSH, and TSH levels in kidney tissue - reduced RONS, LPO, and MDA levels in kidney tissue - reduced NO levels and MPO activity in kidney tissue - reduced IL-1β and TNF-α levels in kidney tissue - increased IL-10 level in kidney tissue	- increased survival rate - decreased serum creatinine and urea levels - decreased caspase-3 and caspase-9 activity - histological: decreased glomerular hypercellularity and preserved kidney architecture	[109]

Abbreviation: Akt—protein kinase B; SERCA2a—sarcoplasmic/endoplasmic reticulum Ca^2+^-ATPase 2a; MDA—malondialdehyde; NO—nitric oxide; MPO—myeloperoxidase; GSH—glutathione; GPx—glutathione peroxidase; GST—glutathione-S-transferase; NF-κB—nuclear factor- κB; CAT—catalase; SOD—superoxide dismutase; miR320—MicroRNA-320; Nrf-2—nuclear factor erythroid 2 related factor 2; BUN—blood urea nitrogen; 3-NT—3-nitrotyrosine; 4-HNE—4-hydroxynonenal; COX-2—cyclooxygenase-2; TNFα—tumor necrosis factor-alpha; p53—protein-53; IL-1β—interleukin 1 beta; IL-6—interleukin 6; TSH—total sulfhydryl group; RONS—reactive oxygen/nitrogen species; LPO—lipid peroxidation; IL-10—interleukin 10.

**Table 5:** Effects of resveratrol on heart failure and non-diabetic chronic kidney disease.

Different Models of Heart Failure				
Dosage of Resveratrol	Model	Anti-Inflammatory and Antioxidant Effects	Beneficial Heart Effects	Reference
Resveratrol (45.51 mg/kg/day–oral for 28 days)	24 rats with angiotensin II-induced cardiopathy	- down-regulated NF-kB expression	- increased left ventricular ejection fraction and fractional shortening - reduced left ventricle wall thickness - mitigated angiotensin II-induced cardiac hypertrophy - reduced interstitial fibrosis - down-regulated ACTA1, ANP, BNP, collagen-I, and collagen-III expression - down-regulated Ang-II/AT1R signal transduction	[118]
Resveratrol (20mg/kg/day–intraperitoneal for 42 days)	95 C57BL/6 rats with left coronary artery ligation-induced cardiopathy	- down-regulated ANP, ICAM-1, MMP-9, FKN, procollagen-I, and procollagen-III expression	- increased survival rate - echocardiography: reduced left ventricular end-diastolic and end-systolic diameters; increased left ventricular fractional shortening - reduced left ventricle infarct size	[115]
Resveratrol (450 mg/kg/day–oral for 14 days)	44 C57B1/6N male rats with transverse aortic constriction pressure-overload-induced heart failure	-	- increased total basal physical activity level - increased metabolic rate - up-regulated insulin signaling in the skeletal muscle	[126]
Pterostilbene (50 mg/kg/day–oral for 56 days)	30 C57BL/6 male rats with transverse aortic constriction-induced heart failure	- up-regulated Sirt1 and GPX4 expression - down-regulated p-GSK-3β expression	- increased left ventricular ejection fraction and fractional shortening - increased cardiac index - decreased left ventricular internal dimensions at systole and diastole - ameliorated cardiac remodeling by reducing cardiac collagen volume fraction	[127]
Resveratrol (15 mg/kg/day–oral for 56 days)	30 Wistar male rats with isoproterenol-induced heart failure	- reduced nitrotyrosine levels - increased Akt-1and GSK-3β levels - reduced phosphorylation of ERK1/2 and p38-MAPK - down-regulated iNOS and COX-2 expression	- reduced serum BNP level - echocardiography: reduced left ventricular wall thickness, end-systolic volume, and systolic-left ventricular inner diameter - histological: decreased interstitial fibrosis	[128]
Resveratrol (22.5 mg/kg/day–oral for 14 days)	48 Sprague-Dawley male rats with left anterior descending artery ligated-induced heart failure	- did not change Sirt1, AMPK and Akt levels - did not change Mn-SOD and GPx1 levels - down-regulated CYP1B1 expression	- echocardiography: increased left ventricular ejection fraction and reduced cardiac remodeling by decreasing left atrial mass and left ventricular end-diastolic dimension - increased exercise capacity - improved cardiac energy metabolism	[129]
Resveratrol (320 mg/kg/day–oral for 14 days)	67 C57B1/6 male rats with transverse aortic constriction pressure-overload-induced heart failure	- restored AMPK activation	- increased survival rate - histological: reduced cardiac fibrosis - down-regulated cardiac hypertrophy gene expression: anf, ska, bnp, β-mhc - improved diastolic function and reduced left atrial volume - improved myocardial insulin sensitivity	[130]
Resveratrol (10 mg/kg/day–oral for 28 days)	18 C57BL6 male rats with transverse aortic constriction pressure-overload-induced heart failure	- significantly reduced left ventricular macrophage and mast cell infiltration - down-regulated 4-HNE and 8-OHdG expression - reduced HIF-1α levels - increased SOD and glutathione activity in left ventricle homogenate	- reduced diastolic-left ventricular internal dimension and diastolic-left ventricular posterior wall thickness - increased left ventricular ejection fraction and fractional shortening - histological: reduced perivascular and interstitial fibrosis	[117]
Resveratrol (50 mg/kg/day–oral for 10 months)	52 C57BL/6J rats with aortic coarctation-induced heart failure	- up-regulated SOD, GSH, GPX4, and SLC7A11 expression	- down-regulated BNP and sST2 expression - increased left ventricular ejection fraction - reduced myocardial edema, hypertrophy, and fibrosis	[119]
Resveratrol (5mg/kg/day–oral for 10 months)	110 Wistar male rats with left coronary artery ligation-induced heart failure	-	Echocardiography - reduced aortic stiffness - improved left ventricular systolic function	[131]
Resveratrol (8mg/kg/day–intraperitoneal for 28 days)	30 Sprague Dawley male rats with abdominal aorta ligated, pressure-overload-induced heart failure	-	- increased left ventricular ejection fraction and fractional shortening - down-regulated BNP mRNA expression - reduced autophagy by down-regulating beclin-1 and lamp-1 expression - increased myocardial ATP level	[132]
Resveratrol (2.5 mg/kg/day–oral for 56 days)	30 Sprague Dawley male rats with left anterior descending artery ligated-induced heart failure	- reduced MDA and TNF-α levels in left ventricular homogenate	- reduced systolic and diastolic left ventricular internal diameter - reduced end-diastolic and end-systolic volumes - increased left ventricular ejection fraction and fractional shortening - histological: reduced collagen levels - reduced BNP levels	[116]
Resveratrol (2.5 mg/kg/day–oral for 112 days)	50 male rats with the left anterior descending artery ligated-induced heart failure	- increased phosphorylation of AMPK - up-regulated Sirt1 expression	- increased survival rate - reduced BNP levels - increased left ventricular ejection fraction	[133]
Resveratrol (100 mg/kg/day–oral for 3 months)	60 patients with heart failure with reduced ejection fraction in a single-center, randomized, double-blind, placebo-controlled study	- significantly reduced IL-6 and IL-1 levels	- significantly reduced galectin-3 and NT-proBNP levels - echocardiography: increased left ventricular ejection fraction, stroke volume; decreased left ventricular end-systolic volume; improved global longitudinal strain - increased exercise capacity - improved respiratory parameters - improved quality of life	[120]
**Different Models of Non-Diabetic Chronic Kidney Disease**				
**Dosage of Resveratrol**	**Model**	**Anti-inflammatory and Antioxidant Effects**	**Beneficial Kidney Effects**	**Reference**
Resveratrol (20 mg/kg/day–oral for 28 days)	116 Sprague Dawley male rats with 5/6 nephrectomy-induced chronic kidney disease	Renal mitochondria - increased MMP and ATP levels - decreased ROS levels - increased complex-I and complex-III activity - increased Sirt1 activity	-	[134]
Resveratrol (100 mg/kg/day–oral for 28 days)	30 C57BL/6 male rats with high-fat diet-induced nephropathy	- decreased TNF-α and IL-6 levels in renal homogenate - increased IL-10 levels in renal homogenate - decreased MDA levels and increased T-SOD and GPx levels in renal homogenate - down-regulated TLR4, MCP-1, CD11c and F4/80 expression	- decreased serum creatinine and BUN levels - histological: reduced kidney hypertrophy, enlargement of the mesangial area, and tubular vacuolization	[123]
Pterostilbene (200 mg/kg/day–oral for 7 days)	20 ICR male rats with a high-adenine diet-induced chronic kidney disease	- histological: reduced accumulation of inflammatory cells such as macrophages and CD68^+^ - down-regulated renal TGF-β and fibronectin expression - up-regulated E-cadherin expression	- reduced serum creatinine, uric acid, and BUN levels, increasing these levels in urine - histological: reduced tubular dilatation and interstitial fibrosis	[122]
Resveratrol (20 mg/kg/day–oral for 14 days)	24 C57BL/6J male rats with unilateral ureteral obstruction-induced nephropathy	- increased SOD levels and decreased MDA and 8-OHdG levels in renal homogenate - down-regulated fibronectin, TGF-β, TNF-α, and ICAM-1 expression - up-regulated Sirt1 expression	-histological: mitigated renal fibrosis by reducing glomerular injury and collagen accumulation	[124]
Resveratrol (20 mg/kg/day–oral for 7 months)	26 male Wistar rats with uninephrectomy-induced chronic kidney disease	- reduced TNF-α, IDO, and IL-1β levels	- reduced serum creatinine and urea levels - reduced proteinuria - insignificantly reduced urine protein-to-creatinine ratio	[135]
Resveratrol (5 mg/kg/day–oral for 84 days)	50 male Wistar rats with 5/6 nephrectomy-induced chronic kidney disease	-	- significantly increased survival rate - reduced proteinuria - reduced serum creatinine, urea, and BUN levels - histological: reduced glomeruli sclerosis and interstitial fibrosis	[121]
Resveratrol (500 mg/day–oral for 28 days)	20 non-dialyzed CKD patients in a randomized, crossover, double-blind trial	- no significant activity on Nrf2 and NF-κB expression - no significant activity on CRP, IL-6, TNF-α, CAT, SOD, GPx activity	- no significant activity on serum creatinine and urea levels	[113]
**Different Models of both Heart Failure and Non-Diabetic Chronic Kidney Disease**				
**Dosage of Resveratrol**	**Model**	**Anti-inflammatory and Antioxidant Effects**	**Beneficial Kidney Effects**	**Reference**
Resveratrol (20 mg/kg/day–oral for 84 days)	12 Sprague–Dawley and wild-type C57BL/6J rats with 5/6-nephrectomy-induced chronic kidney disease	- increased Sirt1 activity in the heart - decreased 8-OHdG level in the heart - up-regulated MnSOD expression in the heart	- reduced serum creatinine and BUN levels - renal histology: mitigated renal injury, such as glomerular hypertrophy and sclerosis, necrosis of tubulointerstitial tissue - reduced fibronectin and collagen-1 levels - ameliorated dilatation and wall thickening of the heart on echocardiography - heart histology: reduced myocyte hypertrophy, perivascular and interstitial fibrosis	[125]

Abbreviation: NF-κB—nuclear factor- κB; ACTA1—skeletal α-actin; ANP—atrial natriuretic peptide; BNP—brain natriuretic peptide; Ang-II—angiotensin-II; AT1R—angiotensin II type 1 receptor; ICAM-1—intercellular adhesion molecule 1; MMP-9—matrix metalloproteinase-9; FKN—fractalkine; Sirt1—silent information regulator 1; GPX4—glutathione peroxidase 4; p-GSK-3β—phosphorylation of glycogen synthase kinase-3β; Akt—protein kinase B; ERK1/2—extracellular-receptor kinases; p38-MAPK—p38 mitogen-activated protein kinase; iNOS—inducible nitric oxide synthase; COX-2—cyclooxygenase-2; AMPK—adenosine monophosphate-activated protein kinase; MnSOD—manganese-containing superoxide dismutase; GPx1—glutathione peroxidase 1; CYP1B1—cytochrome P450 1B1; 4-HNE—4-hydroxynonenal; 5-OHdG—8-hydroxydeoxyguanosine; HIF1α—hypoxia-inducible factor 1-alpha; SOD—superoxide dismutase; GSH—glutathione; SLC7A11—solute carrier family 7 member 11; sST2—soluble suppression of tumorigenicity-2; MDA—malondialdehyde; TNFα—tumor necrosis factor-alpha; IL-6—interleukin 6; IL-1—interleukin 1; MMP—matrix metalloproteinase; ATP—adenosine triphosphate; ROS—reactive oxygen species; IL-10—interleukin 10; BUN—blood urea nitrogen; GPx—glutathione peroxidase; TLR4—toll-like receptor 4; MCP-1—monocyte chemotactic protein 1; CD11c—integrin alpha X; F4/80—adhesion G protein-coupled receptor E1; TGF-β—transforming growth factor beta; IDO—indoleamine-2,3-dioxygenase; Nrf-2—nuclear factor erythroid 2 related factor 2; CRP—C-reactive protein; CAT—catalase.

**Table 6:** Common and distinct mechanisms of action and effects of natural compounds in non-diabetic chronic kidney disease.

Mechanisms/Effects	Arjunolic Acid	Kaempferol	Luteolin	Resveratrol	Shared Pathways in ND-CKD
Antioxidant activity	↗ GSH ↘ MDA, NO, LAP	↗ SOD, CAT, GPx, GST ↘ MDA, iNOS, TBARS, ROS	↗ SOD, CAT, GPx ↘ MDA, H_2_O_2_	↗ SOD, GPx, T-SOD ↘ MDA, ROS	Reduction in oxidative stress and ROS
Anti-inflammatory activity	↘ NF-κB, TNF-α, IL-1β, Kim-1	↘ TNF-α, IL-1β, IL-6, JNK, ERK1/2	↘ TNF-α, IL-1β, COX-2, NF-κB	↗ IL-10 ↘ TNF-α, IL-6	Downregulation of pro-inflammatory cytokines
Antifibrotic effects	↘ TGF-β	↘ TGF-β, α-SMA, collagen I/III	↘ TGF-β, fibronectin, collagen I	↘ TGF-β, fibronectin, collagen I	Suppression of fibrosis and ECM deposition
Apoptosis regulation	↗ Bcl-2 ↘ Caspase-8/9	↘ Bax/Bcl-2, TP53	↗ Bcl-2 ↘ p53, Bax, caspase-3	↗ Sirt1 ↘ Bax	Protection from cell death via apoptotic modulation
Mitochondrial effects	Not prominently described	Not prominently described	Not prominently described	↗ ATP, Sirt1, complex-I/III activity	Enhancement of mitochondrial function (resveratrol-specific)
Renal function improvement	↗ survival rate ↘ creatinine, BUN, urea	↘ creatinine, BUN, tubular necrosis	↘ creatinine, BUN, structural damage	↗ renal histology ↘ creatinine, BUN, proteinuria	Functional and structural nephroprotection

**Table 7:** Common and distinct mechanisms of action and effects of natural compounds in heart failure.

Mechanisms/Effects	Arjunolic Acid	Kaempferol	Luteolin	Resveratrol	Shared Pathways in HF
Antioxidant activity	Not prominently described	↗ SOD, CAT, GPx, GST ↘ MDA	↗ GPx, GST, GSH ↘ MDA, NO	↗ SOD, GPx, GSH ↘ MDA, 4-HNE, 8-OHdG	Reduction in oxidative burden in myocardium
Anti-inflammatory activity	↘ TGF-β	↘ TNF-α, IL-1β, IL-6, NF-κB	↘ TNF-α, IL-1β, NF-κB	↘ TNF-α, IL-6, IL-1, NF-κB	Attenuation of cardiac inflammation
Improvement in cardiac function	↗ PPARα ↗ Ejection fraction ↘ Collagen I/III	↘ Nrf2 ↗ Ejection fraction Maintained SBP ↘ BNP	↗ Ejection fraction and Fractional shortening ↘ LV diameters	↗ Ejection fraction, fractional shortening, and hemodynamics ↘ BNP	Enhancement of systolic and diastolic function
Remodeling and fibrosis inhibition	↘ Collagen I/III	↗ Titin preservation ↘ Myocardial fibrosis	↘ Myocardial apoptosis, fibrosis	↘ Collagen, fibronectin, hypertrophy genes	Structural cardiac protection via antifibrotic actions
Apoptosis regulation	Not prominently described	↗ Nrf2 ↘ NF-κB	↘ Caspase-3, p53, Bax	↗ Akt, Sirt1 ↘ apoptosis, ICAM-1	Cardiomyocyte survival via anti-apoptotic mechanisms
Energy metabolism/Mitochondrial effects	↗ PPARα	Mild or indirect involvement	↗ SERCA2a, Akt	↗ AMPK, Sirt1, ATP, insulin sensitivity	Improved cardiac bioenergetics and metabolic efficiency

## Data Availability

Not applicable.
